# Longitudinal Associations Between Cognitive Deficits in Childhood and Psychopathological Symptoms in Adolescence and Young Adulthood

**DOI:** 10.1001/jamanetworkopen.2021.4724

**Published:** 2021-04-07

**Authors:** Isabel Morales-Muñoz, Rachel Upthegrove, Pavan K. Mallikarjun, Matthew R. Broome, Steven Marwaha

**Affiliations:** 1 Department of Public Health Solutions, Finnish Institute for Health and Welfare, Helsinki, Finland; 2 Institute for Mental Health, School of Psychology, University of Birmingham, Birmingham, United Kingdom; 3 Early Intervention Service, Birmingham Women’s and Children’s NHS Foundation Trust, Birmingham, United Kingdom; 4 Specialist Mood Disorders Clinic, Zinnia Centre, Birmingham, United Kingdom

## Abstract

**Importance:**

Cognitive deficits are core features of mental disorders and are important in predicting long-term prognosis. However, it is still unknown whether individual patterns of cognitive deficits predate specific mental disorders.

**Objective:**

To investigate the specificity of the associations of attention, working memory, and inhibition in childhood with borderline personality disorder (BPD), psychosis, depression, and hypomania in adolescence and young adulthood.

**Design, Setting, and Participants:**

This cohort study obtained data from the Avon Longitudinal Study of Parents and Children in the United Kingdom. All pregnant women resident in Avon, United Kingdom, with an expected date of delivery from April 1, 1991, and December 31, 1992, were eligible. Data analysis was conducted from April 1 to September 30, 2020. The sample initially comprised 13 988 participants who were alive at 1 year of age. For this study, data were available for 6333 individuals reporting on any psychopathological measure at ages 11 to 12 years, 4903 individuals at ages 17 to 18 years, and 2963 individuals at 22 to 23 years.

**Exposures:**

Sustained attention, selective attention, and attentional control were assessed with the Test of Everyday Attention for Children at age 8 years, and working memory and inhibition were assessed at age 10 years with the Counting Span Task and the stop-signal paradigm, respectively.

**Main Outcomes and Measures:**

Symptoms of BPD were assessed at ages 11 to 12 years, psychotic experiences and depression were examined at ages 17 to 18 years, and hypomania was examined at ages 22 to 23 years.

**Results:**

Among 5315 individuals included in the statistical analysis, 2551 (48.0%) were male and 2764 (52.0) were female. Higher sustained attention at 8 years was associated with decreased risk of BPD symptoms at ages 11 to 12 years (adjusted odds ratio [aOR], 0.964; 95% CI, 0.933-0.996; *P* = .03), better performance on inhibition at age 10 years with decreased risk of psychotic experiences at ages 17 to 18 years (aOR, 0.938; 95% CI, 0.890-0.989; *P* = .02), higher sustained attention at age 8 years with decreased risk of depressive symptoms at ages 17 to 18 years (aOR, 0.969; 95% CI 0.938-0.9997; *P* = .048), and better performance in working memory at age 10 years with decreased risk of hypomania symptoms at ages 22 to 23 years (aOR, 0.694; 95% CI, 0.529-0.911; *P* = .008). After controlling for potential psychopathological overlay, all the associations remained, except for working memory and hypomania. Higher sustained attention at age 8 years was associated with decreased risk of BPD symptoms at ages 11 to 12 years (β = −0.05; *P* < .001) and of depression at ages 17 to 18 years (β = −0.03; *P* = .04), and better performance in inhibition at age 10 years was associated with decreased risk of psychotic experiences at ages 17 to 18 years (β = −0.03; *P* = .04).

**Conclusions and Relevance:**

These findings suggest that specific cognitive deficits in childhood are distinctively associated with different psychopathological symptoms in young people. Furthermore, these results suggest the potential of early cognitive interventions in childhood as a way of modifying or attenuating risk for subsequent psychopathological symptoms.

## Key Points

**Question:**

Is there an association between cognition in childhood and mental disorders in adolescence and young adulthood?

**Findings:**

In this cohort study of 5315 individuals from the Avon Longitudinal Study of Parents and Children, 3 childhood cognitive factors were associated with subsequent psychopathological symptoms in young people, and a specificity in these associations was observed. Higher sustained attention at age 8 years was associated with decreased risk of borderline personality disorder symptoms at ages 11 to 12 years and depressive symptoms at ages 17 to 18 years; better working memory at age 10 years was associated with decreased risk of hypomania symptoms at ages 22 to 23 years, and better performance in inhibition at age 8 years was associated with decreased risk of psychotic experiences at ages 17 to 18 years.

**Meaning:**

These findings suggest that specific cognitive deficits should be considered as targetable endophenotypes in the prediction and intervention for specific mental disorders, such as borderline personality disorder, depression, and psychosis.

## Teaser

This cohort study investigates the associations between cognitive measures in childhood and psychopathological conditions in adolescence and adulthood.

## Introduction

Mental disorders are associated with significant disease burden globally, and at least 10% of children and adolescents worldwide have a mental disorder.^1^ Among mental disorders diagnosed in adults, 75% have an onset in childhood and adolescence.^2^ Bipolar disorder, depression, and psychosis commonly emerge during adolescence and continue in young adulthood.^3^ Their emergence is probably associated with anomalies of adolescent maturational processes associated with psychosocial, biological, or environmental factors.^4^ Therefore, it is crucial to study the onset of mental disorders at these early stages and investigate which risk factors predate onset and in what ways they are associated.

Cognitive problems are core features of mental disorders, such as psychosis^5^ and mood disorders.^6^ Deficits in cognitive function, ranging from decreased attention and working memory to disrupted social cognition and language, are common in individuals with psychiatric disorders and severely compromise quality of life, including social and professional integration.^7^ Cognition develops in childhood alongside the appearance of psychopathological symptoms.^8^ However, cognitive difficulties may appear before unequivocal symptoms associated with mood or behavior and could potentially predate these by several years. For instance, cognitive deficits in childhood are associated with subsyndromal psychotic experiences in adulthood.^9^ However, to what extent cognitive impairments in childhood precede a range of mental disorders in adolescence and young adulthood is unknown. Determining which cognitive domains are associated with specific mental disorders is complicated by comorbidity, especially in youth populations.

As far as we are aware, existing studies have not examined the prospective and specific associations of childhood cognition with different forms of psychopathological symptoms in young people. It is essential to do so to understand how mental disorders develop, whether there are specific pathways associated with the development of specific mental disorders, and to what extent this development could be changed. In this study, we aimed, first, to add to our previous work on the risk factors associated with various psychopathological conditions^10,11^ by using longitudinal data to investigate the associations of childhood attention, working memory, and inhibition with subsequent psychopathological symptoms found in borderline personality disorder (BPD), psychosis, depression, and hypomania. These are some of the most common psychopathological dimensions found among young people. Second, we aimed to investigate whether individual cognitive domains in childhood distinctively associate with specific psychopathological symptoms in young people. We hypothesized that working memory and inhibition would be associated with psychotic experiences, given that executive function is one of the most commonly observed deficits among individuals with schizophrenia through various stages of the disease,^12^ and that attention would be associated with depression,^13^ BPD,^14^ and hypomania.^15^

## Methods

Ethical approval for this cohort study was obtained from the Avon Longitudinal Study of Parents and Children (ALSPAC) Law and Ethics Committee and local research ethics committees. Informed consent for the use of data collected via questionnaires and clinics was obtained from participants following the recommendations of the ALSPAC Ethics and Law Committee at the time. This study followed the Strengthening the Reporting of Observational Studies in Epidemiology (STROBE) reporting guideline.

### Participants

The UK birth cohort ALSPAC examines the determinants associated with development, health, and disease during childhood and at later ages.^16,17^ Pregnant women residing in Avon, UK, with expected dates of delivery from April 1, 1991, to December 31, 1992, were invited to take part in the study. The ALSPAC website contains details of all the data available.^18^ Further details of this cohort are described in the eAppendix in the Supplement.

### Measures

#### Cognitive Domains

Cognitive measures were obtained from the Test of Everyday Attention for Children (TEA-CH)^19^ at age 8 years and computerized versions of the counting span task^20^ and the stop-signal paradigm^21^ at age 10 years. These 3 tests focus on attention, working memory, and inhibitory control, respectively, which are the 3 cognitive domains most commonly observed in psychopathological conditions.^22^

To measure attention, 3 tasks were selected from TEA-CH: the sky search task, for selective attention; the sky search dual task, for sustained attention; and the opposite-worlds task, for attentional control and switching. In the sky search task, participants were asked to identify pairs of identical “spacecraft” from a page of visually similar stimuli while ignoring all distracting stimuli. Time and accuracy were recorded, and a motor control test was performed. An age-corrected normative score was calculated based on the manual instructions^19^ and was adjusted for motor control. The sky search dual task, which has been found to be associated with sustained attention factors,^19^ follows the same procedure as the sky search task, with the addition of simultaneously presented auditory stimuli. Participants were requested to count while performing the sky search task. Normative scores based on time and errors were calculated and used as the measure for analysis.^19^ The opposite-worlds task involves 2 conditions: first, participants followed digits printed on a handout and stated the numbers out loud; second, participants had to inhibit the predominant response and state “1” when presented with the digit 2, and “2” when presented with the digit 1. Errors resulted in a time penalty, and normative scores were calculated based on this,^19^ which was used as a measure.

Visual working memory at age 10 years was assessed using a computerized version of the counting span task.^20^ Participants were shown a number of red and blue dots. After seeing each set, participants were asked to recall the number of red dots in the order they were presented within that set. The working memory span measure was calculated as the number of correctly recalled sets weighted by the number of screens within each set.

Inhibitory control at age 10 years was measured with the stop signal paradigm^21^ using the procedure outlined by Handley et al.^23^ Two types of trials were performed. In primary trials, participants were asked to fixate on a small smiley face presented in the center of the screen. An X or O was presented, and participants had to press the button as quickly as possible. In stop signal trials, participants were asked to do the same task but with an audible beep occurring after presentation of the X or O on certain trials. Participants were told to avoid pressing the button when the beep was sounded. The number of trials in which the participant correctly stopped when the stop signal occurred 150 milliseconds before participant's mean reaction time was used as a measure.

#### Outcomes

##### BPD Symptoms at Ages 11 to 12 Years

We assessed BPD psychopathological symptoms using a face-to-face semistructured interview: the UK Childhood Interview for *Diagnostic and Statistical Manual of Mental Disorders* (Fourth Edition) BPD.^24^ The derived dichotomous outcome was based on a 2019 study,^25^ representing the very frequent or repeated occurrence of 5 or more BPD symptoms, consistent with the criteria used in diagnosis.

##### Psychotic Experiences at Ages 17 to 18 Years

The Psychosis-Like Symptoms Interview is a semistructured face-to-face interview^26^ with 12 questions about psychotic experiences. Participants were asked about their experiences since their 12th birthdays. We coded the presence of at least 1 definite psychotic symptom not associated with sleep or fever.

##### Depressive Symptoms at Ages 17 to 18 Years

Symptoms of depression were assessed using the validated Revised Clinical Interview Schedule (CIS-R).^27^ The CIS-R was used to establish the severity of core symptoms of depression. Following a 1992 study,^11^ we constructed a dichotomous variable.

##### Hypomania Symptoms at Ages 22 to 23 Years

Hypomania symptoms were defined using the Hypomania Checklist, a self-report measure of lifetime experience of manic symptoms.^28^ Participants were asked to consider a time when they were in a “high or hyper” state and to endorse a number of statements about their emotions, thoughts, and behaviors at that time. We defined lifetime history of clinically relevant hypomanic symptoms in line with a 2016 study.^29^

#### Covariates

Family adversity in childhood was assessed in the mother using the Family Adversity Index (FAI) during pregnancy (using the long index), when the child was age 2 years (using the long index), and when the child was age 4 years (using the short index). The FAI comprises 18 items (ie, long index) on childhood adversity and socioeconomic status, including maternal mental health. The short index excludes social, practical, and financial support. If an adversity item was reported, 1 point was given. The total FAI scores for the 3 time points were summed and entered into the analysis as a continuous variable, in line with recommended use^30^ and a 2012 study.^31^

Childhood physical and sexual abuse were reported by the mother when children were ages 1.5 years, 3.5 years, 4.8 years, 5.8 years, and 6.8 years. Consistent with a 2012 study,^32^ abuse was classified as present if sexual or physical abuse was reported at any time point.

Emotional temperament was reported by the mother using the Carey Temperament Scale (CTS)^33^ when children were age 2 years. The Mood and Intensity subscales of the CTS were chosen because they map most closely onto emotional temperament.^34^ Total scores from the mood and intensity scales were summed.

Being bullied in childhood was reported by the mother when the child was age 8 years from a single item of the Strengths and Difficulties Questionnaire:^35^ “child is picked on or bullied by other children.” If the response was “somewhat applies” or “certainly applies,” the child was considered to have experienced bullying.

Child sex and gestational age were included as confounders. All confounders were selected based on the association that they have with mental health, following previous research.^36,37,38,39,40^

### Statistical Analysis

Among 13 988 participants in the original sample, 7987 participants (57.1%) were lost to follow-up at ages 11 to 12 years. Therefore, we conducted logistic regressions to identify factors associated with attrition. Adolescents and young adults lost to attrition were more often boys, had higher scores in family adversity, and reported lower levels of childhood abuse (eTable 1 and eTable 2 in the Supplement). Using the variables associated with selective dropout as predictors, we fitted a logistic regression model (nonresponse vs response outcome) to determine weights for each individual using the inverse probability of response. We used this weighting variable in the logistic regression analyses to control for any influence of selection bias associated with dropout.

A multistage analysis plan was developed. The first stage was conducted using SPSS statistical software version 25 (IBM) to ascertain the associations between cognitive measures in childhood and psychopathological symptoms in young people. We ran logistic regression analyses to assess the associations between cognitive domains (ie, selective attention, sustained attention, attentional control, inhibition, and working memory) in childhood and subsequent pathological outcomes (ie, BPD symptoms, psychotic experiences, depression, and hypomania). Cognitive factors were treated as continuous variables, while psychopathological outcomes were treated as dichotomous, with categories that would indicate clinically relevant psychopathological symptoms. In model 1 we tested unadjusted associations. In model 2, we controlled for child’s sex, family adversity, childhood abuse, child’s emotional temperament, being bullied in childhood, and gestational age. In both models, all the explanatory variables were included together.

In the second stage, we conducted a path analysis in SPSS Amos statistical software version 27 (IBM), with maximum likelihood estimation to investigate the pathways to different psychopathological outcomes and whether any associations found were independent of associations between the outcomes (eg, due to comorbidity). We included as independent variables only those cognitive variables with significant associations in model 2 (ie, sustained attention, working memory, and inhibition). Finally, we controlled for all confounders and for the associations among all the outcomes. Missing data were dealt with using the full information maximum likelihood method.^41^ We used bootstrapped bias-corrected confidence intervals and *P* values for assessing the significance of the standardized effects. *P* values were 1-sided, and statistical significance was set at *P* < .05. Data analysis was conducted from April 1 to September 30, 2020.

## Results

Among 13 988 individuals in the sample alive at age 1 year, data were available on 6333 individuals reporting on any psychopathological measure at ages 11 to 12 years (3277 girls [51.7%] and 3056 boys [48.3%]), 4903 individuals at ages 17 to 18 years (2792 female participants [56.9%] and 2111 male participants [43.1%]), and 2963 individuals at ages 22 to 23 years (1912 women [64.7%] and 1051 men [35.3%]). Among 5315 individuals who had cognition measures at ages 11 to 12 years in childhood and were included the statistical analysis, 2551 were female (52.0%) and 2551 were male (48.0%); among 5089 individuals, mean (SD) gestational age was 39.42 (1.87) weeks (Table 1). A flowchart of the study sample can be found in eFigure in the Supplement. Table 1 shows the frequencies and descriptive values of sociodemographic, cognitive, and psychopathological variables. A description of the symptoms of greatest relevance for BPD, psychosis, and hypomania is presented in eTable 3 in the Supplement, and eTable 4 in the Supplement presents the descriptive values of the cognitive variables for each outcome.

**Table 1.** Characteristics of Participants Included in Statistical Analyses

Characteristic (N = 5315)	Value
Sex, No (%) (n = 5315)	
Male	2551 (48.0)
Female	2764 (52.0)
Gestational age in wk, mean (SD) (n = 5089)	39.42 (1.87)
Emotional temperament at age 2 y, mean (SD) (n = 4688)^a^	39.16 (8.31)
Childhood abuse, No. (%) (n = 5315)	
Yes	559 (11.2)
No	4436 (88.8)
Family adversity, mean (SD) (n = 4752)^b^	3.67 (3.81)
Being bullied at age 8 y, No. (%) (n = 4371)	
Yes	728 (16.7)
No	3643 (83.3)
Normative score at age 8 y, mean (SD)^c^	
Selective attention (n = 5315)	8.72 (2.18)
Sustained attention (n = 5187)	7.71 (3.70)
Attentional control (n = 5268)	18.34 (1.55)
Age 10 y, mean (SD)	
Working memory span score (n = 4772)^d^	3.44 (0.84)
Inhibition (n = 4751)^e^	12.07 (3.06)
Ages 11-12 y	
BPD total symptoms, mean (SD) (n = 5315)	1.17 (1.76)
Individuals with or without BPD symptoms, No. (%) (n = 5315)	
Yes	373 (7.0)
No	4942 (93.0)
Ages 17-18 y	
No. of Psychotic symptoms, mean (SD) (n = 3249)	0.06 (0.40)
Individuals with or without psychotic experiences, No (%), (n = 3249)	
Yes	135 (4.2)
No	3114 (95.8)
Depressive symptoms total score, mean (SD) (n =3177)^f^	3.18 (3.91)
Individuals with or without depressive symptoms, No. (%) (n = 3168)	
Yes	332 (10.5)
No	2836 (89.5)
Ages 22-23 y	
Hypomania symptoms total score, mean (SD) (n = 1958)^g^	15.20 (6.06)
Individuals with or without hypomania symptoms, No. (%) (n = 1931)	
Yes	66 (3.4)
No	1865 (96.6)

Abbreviation: BPD, borderline personality disorder.

^a^ Total scores from the mood and intensity scales from the Carey Temperament Scale were summed.

^b^ The total Family Adversity Index scores for 3 time-points (ie, during pregnancy, age 2 years, and age 4 years) were summed.

^c^ An age-corrected normative score was calculated based on the manual instructions from the Test of Everyday Attention for Children.

^d^ The working memory span measure was calculated as the number of correctly recalled sets weighted by the number of screens within each set.

^e^ The number of correct trials inhibited when the stop signal occurred 150 ms before participant's mean reaction time was used as the measure.

^f^ The validated Revised Clinical Interview Schedule was used to establish the severity of core symptoms of depression.

^g^ Participants were asked to consider a time when they were in a “high or hyper” state and endorse a number of statements about their emotions, thoughts, and behaviors at that time.

**Figure 1.**
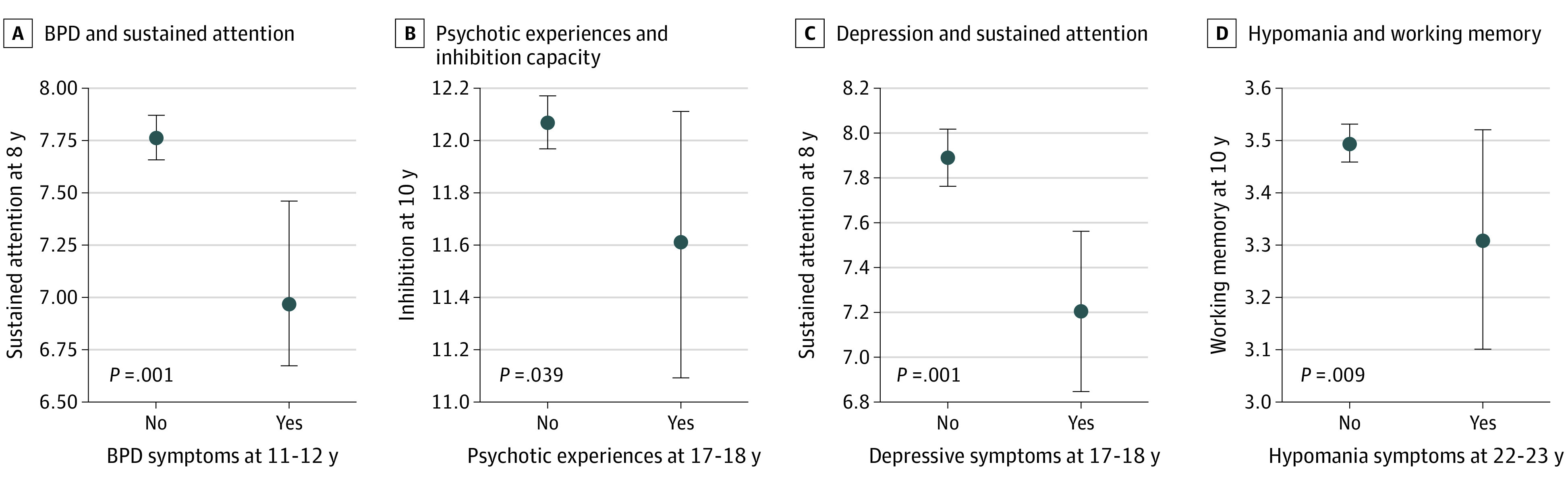
Mean Differences Between Cognitive Functioning in Childhood and Psychopathological Symptoms in Adolescence and Young Adulthood BPD indicates borderline personality disorder; error bars, 95% of values that are less than 2 SEs away from the mean.

Table 2 shows the associations between each cognitive measure in childhood and subsequent psychopathological symptoms. As can be seen from the table, the variance increased in the adjusted model for all outcomes. In the adjusted analysis (ie, model 2), associations were found between higher sustained attention at age 8 years and decreased risk of BPD symptoms at ages 11 to 12 years (adjusted odds ratio [aOR], 0.964; 95% CI, 0.933-0.996; *P* = .03), better performance on inhibition at age 10 years and decreased risk of psychotic experiences at ages 17 to 18 years (aOR, 0.938; 95% CI, 0.890-0.989; *P* = .02), higher sustained attention at age 8 years and decreased risk of depressive symptoms at ages 17 to 18 years (aOR, 0.969; 95% CI, 0.938-0.9997; *P* = .048), and better performance in working memory at age 10 years and decreased risk of hypomania symptoms at ages 22 to 23 years (aOR, 0.694; 95% CI, 0.529-0.911; *P* = .008). In Figure 1, a visualization of the mean significant differences in cognitive scores for each psychopathological outcome is presented.

**Table 2.** Logistic Regression Analyses Between Cognitive Measures and Psychopathological Symptoms in Adolescence

	Model 1^a^				Model 2^b^			
	Participants, No.	*R^2^*	OR (95% CI)	*P* value	Participants, No.	*R^2^*	OR (95% CI)	*P* value
**BPD symptoms at ages 11-12 y**								
Age 8 y								
Selective attention	4042	0.004	0.974 (0.926-1.025)	.32	3519	0.014	0.977 (0.924-1.034)	.42
Sustained attention	4042	0.004	0.959 (0.931-0.988)	.006	3519	0.014	0.964 (0.933-0.996)	.03
Attentional control	4042	0.004	0.952 (0.891-1.017)	.15	3519	0.014	0.942 (0.877-1.012)	.10
Age 10 y								
Working memory	4042	0.004	0.894 (0.782-1.023)	.13	3519	0.014	0.922 (0.796-1.067)	.28
Inhibition	4042	0.004	0.981 (0.948-1.016)	.29	3519	0.014	0.983 (0.946-1.022)	.39
**Psychotic experiences at ages 17-18 y**								
Age 8 y								
Selective attention	2892	0.003	0.932 (0.864-1.004)	.06	2552	0.011	0.922 (0.850-1.001)	.05
Sustained attention	2892	0.003	0.992 (0.950-1.037)	.74	2552	0.011	0.996 (0.949-1.046)	.88
Attentional control	2892	0.003	0.955 (0.863-1.056)	.37	2552	0.011	0.931 (0.840-1.032)	.17
Age 10 y								
Working memory	2892	0.003	0.953 (0.788-1.152)	.62	2552	0.011	1.033 (0.837-1.273)	.76
Inhibition	2892	0.003	0.959 (0.912-1.008)	.10	2552	0.011	0.938 (0.890-0.989)	.02
**Depressive symptoms at ages 17-18 y**								
Age 8 y								
Selective attention	2831	0.005	0.979 (0.931-1.029)	.40	2718	0.028	0.978 (0.928-1.031)	.41
Sustained attention	2831	0.005	0.955 (0.927-0.983)	.002	2718	0.028	0.969 (0.938-1.000)	.048
Attentional control	2831	0.005	0.945 (0.879-1.016)	.13	2718	0.028	0.968 (0.896-1.047)	.42
Age 10 y								
Working memory	2831	0.005	1.000 (0.880-1.135)	1.00	2718	0.028	0.999 (0.874-1.143)	.99
Inhibition	2831	0.005	1025 (0.989-1.063)	.17	2718	0.028	1.019 (0.982-1.058)	.32
**Hypomania symptoms at ages 22-23 y**								
Age 8 y								
Selective attention	1746	0.006	1.067 (0.957-1.190)	.24	1574	0.009	1.071 (0.957-1.199)	.24
Sustained attention	1746	0.006	0.996 (0.932-1.065)	.91	1574	0.009	0.974 (0.911-1.041)	.44
Attentional control	1746	0.006	1.081 (0.865-1.350)	.49	1574	0.009	1.068 (0.857-1.332)	.56
Age 10 y								
Working memory	1746	0.006	0.684 (0.524-0.892)	.005	1574	0.009	0.694 (0.529-0.911)	.008
Inhibition	1746	0.006	1.092 (1.001-1.191)	.06	1574	0.009	1.087 (0.994-1.188)	.07

Abbreviations: BPD, borderline personality disorder; OR, odds ratio; R^2^, Cox and Snell *R^2^*.

^a^ Model 1 had no covariates.

^b^ Model 2 was adjusted for sex, childhood abuse, Family Adversity Index score, emotional temperament, being bullied in childhood, and gestational age.

**Figure 2.**
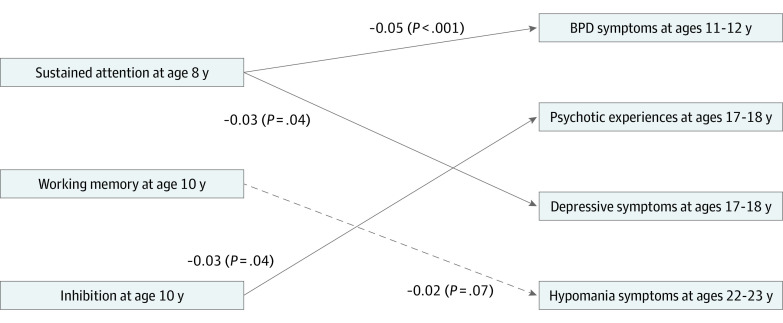
Path Diagram of Direct Associations in the Final Model Solid arrows indicate significant pathways; dashed arrows, nonsignificant modeled pathways; BPD, borderline personality disorder. Pathways of the covariates with independent and dependent variables and pathways between the dependent variables are not shown for clarity. Covariates included in this path analyses were sex, childhood abuse, family adversity, gestational age, emotional temperament, and being bullied in childhood.

In path analysis, we found that model fit indices indicated excellent model fit (χ^2^ = 7.99; *P* = .434; root mean square error of approximation, 0; comparative fit index, 1.00). Consistent with adjusted logistic regression analysis, higher sustained attention at age 8 years was associated with decreased risk of BPD symptoms at ages 11 to 12 years (β = −0.05; *P* < .001) and of depressive symptoms at ages 17 to 18 years (β = −0.03; *P* = .04). Furthermore, better performance in inhibition at age 10 years was associated with decreased risk of psychotic experiences at ages 17 to 18 years (β = −0.03; *P* = .04). However, this path analysis model did not find a significant association between working memory at age 10 years and hypomania at ages 22 to 23 years. Direct associations are shown in Figure 2. The associations between the dependent variables from the path analysis are described in eTable 5 in the Supplement. The significant direct associations between the covariates and the dependent variables are reported in eTable 6 in the Supplement.

## Discussion

To our knowledge, this is the first longitudinal cohort study to explore the extent of specific associations between cognitive factors in childhood and psychopathological domains in young people. We identified 3 cognitive factors associated with subsequent psychopathological symptoms, with some specificity. Our hypothesis that executive function deficits would be associated with psychotic experiences, while attention deficits would be associated with symptoms of depression, BPD, and hypomania was partially confirmed. Supporting our hypotheses, we found that higher sustained attention at age 8 years was associated with decreased risk of BPD symptoms at ages 11 to 12 years and decreased risk of depressive symptoms at ages 17 to 18 years, while better performance in inhibition at age 10 years was associated with decreased risk of psychotic experiences at ages 17 to 18 years. Contrary to our hypothesis, better performance in working memory at age 10 years was associated with decreased risk of hypomania symptoms at ages 22 to 23 years. When we controlled for the potential psychopathological overlay, all the associations remained, except for the association between working memory and hypomania.

First, our finding of an association between sustained attention at age 8 years and risk of BPD symptoms at ages 11 to 12 years is consistent with the finding that sustained attention deficits in adult patients with BPD are associated difficulties in therapy adherence.^42^ Furthermore, results from a 2014 study^43^ suggest the existence of a significant association between adult BPD and history of childhood attention-deficit/hyperactivity disorder (ADHD) symptoms, indicating that ADHD could represent a risk factor associated with BPD. This suggests that sustained attention deficits are associated with BPD given that they are also a key feature of ADHD.^44^

Concerning the associations between childhood sustained attention deficits and depressive symptoms in young people, our findings are consistent with the results of a 2004 study^13^ that found that these deficits can be considered a marker associated with increased risk for major depression. Although impairments in a wide range of cognitive areas have been reported in patients with depression,^45^ our findings emphasize the relevance of sustained attention deficits in childhood preceding the onset of depressive symptoms in young adulthood.

Second, our finding that better performance on inhibition at age 10 years was associated with psychotic experiences in young adulthood, while working memory was not, does not full support our initial hypothesis. However, inhibition is one of the key processes that regulate working memory^46^ and is considered a core component of executive functions.^47^ Therefore, our findings support the notion that deficits in one of the key components of executive functions (ie, inhibition) occurring in childhood precede the onset of later psychotic experiences, and our results are consistent with findings that inhibitory control deficits are common in psychotic disorders.^48^

Third, our finding that better performance in working memory at age 10 years was associated with decreased risk of hypomania symptoms at ages 22 to 23 years is similar to findings on psychotic disorders, in which working memory deficits are often reported in patients with bipolar disorder. Interestingly, the manic/hypomanic bipolar disorder subgroup seems to report greater cognitive impairment in working memory compared with the depressive, mixed, and euthymic subgroup.^49^ However, when we controlled for potential psychopathological comorbidity, the association between working memory and hypomania symptoms disappeared. An explanation might be that psychotic-like symptoms occur during the manic/hypomanic phase of bipolar disorder, and rates of depression are significantly increased among people with mania. Therefore, the associations of cognitive impairments with subsequent hypomanic psychopathological symptoms may be clouded by this other psychopathological condition. However, to our knowledge, no previous research has examined the prospective associations between cognition and hypomanic symptoms, and thus further studies are required.

To date, it has been unclear not only to what extent cognitive deficits in childhood may be associated with subsequent psychopathological symptoms, but also the extent to which these associations may show some element of specificity. These associations could have distinct mechanisms based on neurochemical or regional brain substrates, although this remains to be explored further in neuroimaging studies. More specifically, cognitive deficits and psychopathological symptoms may be associated with shared neural substrates, with cognitive deficits being an early manifestation and psychopathological symptoms a late manifestation associated with those substrates.

**Figure 3.**
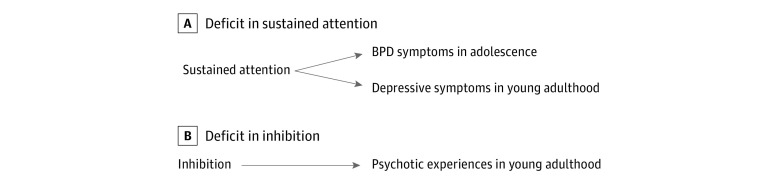
Specific Associations Between Cognitive Domains and Mental Disorders in Adolescence and Young Adulthood BPD indicates borderline personality disorder. This figure presents specific associations that remained after conducting the path analyses.

Our novel findings contribute to the scarce knowledge about the specificity of cognitive impairment across psychopathological dimensions and provide new insights on the potential for development of specific intervention and prevention strategies focused on young people. These strategies could be targetable endophenotypes in the intervention of specific mental disorders, such as BPD, depression, and psychosis (Figure 3). The study results contradict the current prevailing view that psychopathological conditions are best predicted by a general factor *p*,^50^ which challenges the identification of disease causes that confer differential risk to 1 psychiatric disorder but not another. This study has several strengths, including the large population-based sample, longitudinal design, and ability to explore a variety of psychopathological outcomes in young people.

### Limitations

This study has several limitations. First, we were able to control for only known and assessed confounders. Second, measures of cognitive functioning in adolescence and young adulthood were not available, so we do not know whether they were persistent. Furthermore, given that cognitive measures were not available at time points similar to those when the outcomes were assessed, we were not able to account for the comorbidity between cognition and psychopathological outcomes. Third, as is usual in birth cohort studies, the attrition rate was significant. However, we used procedures to ensure the representativeness of our results. Fourth, replication of these results in an independent cohort would strengthen the findings reported in this study. However, we are not aware of any current cohort study with the same measures and the same time points, which would be essential for replication of the findings. Fifth, we did not correct for multiple testing for the main analyses because this study focused on exploratory analysis of multiple separate hypotheses, as opposed to repeated analyses of a single hypothesis.^51^

## Conclusions

This cohort study’s findings support the notion that specific cognitive deficits in childhood are distinctively associated with individual prospective psychopathological problems in young people. Deficits in sustained attention in childhood precede the development of BPD symptoms at ages 11 to 12 years and depression at ages 17 to 18 years, which is probably due to the association between the 2 disorders,^52^ while inhibition impairments in childhood are associated with psychotic experiences in young adulthood. These results highlight the important association between cognitive deficits in childhood and the development of certain mental disorders in young people and suggest the existence of specific prospective associations rather than a generalized impairment. Furthermore, our results suggest that cognition could hypothetically be a target for prevention strategies, though determining the feasibility and nature of such strategies requires further investigation. This study’s results substantially add to previous findings of associations between childhood cognitive deficits and later mental health problems. This study suggests that there are longitudinal associations between childhood cognition and a range of subsequent psychopathological conditions, as well as specific independent pathways in these prospective associations.

## Notes

Supplement. **eAppendix.** Further Details of Avon Longitudinal Study of Parents and Children Cohort **eTable 1.** Differences in Sociodemographic and Clinical Variables Between Nonparticipants and Participants, Ages 11 to 12 Years and 17 to 18 Years **eTable 2.** Differences in Sociodemographic and Clinical Variables Between Nonparticipants and Participants, Ages 22 to 24 Years **eTable 3.** Symptoms of Greatest Relevance for Borderline Personality Disorder, Psychosis, and Hypomania **eTable 4.** Descriptive Values of Cognitive Measures in Psychopathological Outcomes **eTable 5.** Associations Between Dependent Variables in Path Analysis **eTable 6.** Significant Direct Associations Between Covariates and Dependent Variables in Path Analyses **eFigure.** Flowchart of Avon Longitudinal Study of Parents and Children Study Participants
