# Supplementary material for: Longitudinal Associations Between Cognitive Deficits in Childhood and Psychopathological Symptoms in Adolescence and Young Adulthood
Source: JAMA Netw Open. 2021 Apr 7;4(4):e214724. doi: 10.1001/jamanetworkopen.2021.4724 (PMC8027911; doi:10.1001/jamanetworkopen.2021.4724)
Supplement: Supplement. — eAppendix. Further Details of Avon Longitudinal Study of Parents and Children Cohort eTable 1. Differences in Sociodemographic and Clinical Variables Between Nonparticipants and Participants, Ages 11 to 12 Years and 17 to 18 Years eTable 2. Differences in Sociodemographic and Clinical Variables Between Nonparticipants and Participants, Ages 22 to 24 Years eTable 3. Symptoms of Greatest Relevance for Borderline Personality Disorder, Psychosis, and Hypomania eTable 4. Descriptive Values of Cognitive Measures in Psychopathological Outcomes eTable 5. Associations Between Dependent Variables in Path Analysis eTable 6. Significant Direct Associations Between Covariates and Dependent Variables in Path Analyses eFigure. Flowchart of Avon Longitudinal Study of Parents and Children Study Participants [file jamanetwopen-e214724-s001.pdf]

Supplemental Online Content

Morales-Muñoz I, Upthegrove R, Mallikarjun PK, Broome MR, Marwaha S. Longitudinal associations between cognitive deficits in childhood and psychopathological symptoms in adolescence and young adulthood. *JAMA Netw Open*. 2021;4(4):e214724. doi:10.1001/jamanetworkopen.2021.4724

eAppendix. Further Details of Avon Longitudinal Study of Parents and Children Cohort

eTable 1. Differences in Sociodemographic and Clinical Variables Between Nonparticipants and Participants, Ages 11 to 12 Years and 17 to 18 Years

eTable 2. Differences in Sociodemographic and Clinical Variables Between Nonparticipants and Participants, Ages 22 to 24 Years

eTable 3. Symptoms of Greatest Relevance for Borderline Personality Disorder, Psychosis, and Hypomania

eTable 4. Descriptive Values of Cognitive Measures in Psychopathological Outcomes

eTable 5. Associations Between Dependent Variables in Path Analysis

eTable 6. Significant Direct Associations Between Covariates and Dependent Variables in Path Analyses

eFigure. Flowchart of Avon Longitudinal Study of Parents and Children Study Participants

This supplemental material has been provided by the authors to give readers additional information about their work.

© 2021 Morales-Muñoz I et al. *JAMA Network Open.*

eAppendix. Further Details of Avon Longitudinal Study of Parents and Children Cohort

The initial number of pregnancies enrolled was 14541 (for these at least one questionnaire was returned or a "Children in Focus" clinic had been attended by 19/07/99). Of these initial pregnancies, there was a total of 14676 foetuses, resulting in 14062 live births and 13988 children who were alive at 1 year of age. When the oldest children were approximately 7 years of age, an attempt was made to bolster the initial sample with eligible cases who had failed to join the study originally. As a result, in our study, as some variables were collected from the age of seven onwards there were data available for more than the 14541 pregnancies mentioned above.

© 2021 Morales-Muñoz I et al. *JAMA Network Open.*

eTable 1. Differences in Sociodemographic and Clinical Variables Between Nonparticipants and Participants, Ages 11 to 12 Years and 17 to 18 Years

**11-12 years old time point**							
	**Non-participating group in the study**		**Participating group in the study**		**Non-participants vs participants**		
	Mean	*SD*	*Mean*	*SD*	*OR (95% CI)*	*p*	*t-test (t)*
Gestational age (in weeks)	37.61	6.91	39.44	1.85	0.99 (0.97, 1.02)	0.760	20.09
Family Adversity score	5.01	4.64	3.82	3.92	0.95 (0.94, 0.96)	<0.001	-14.08
Emotional Temperament score	39.78	8.67	39.19	8.29	0.99 (0.99, 1.03)	0.328	-3.647
	**Non-participating group in the study**		**Participating group in the study**				
	N	*%*	*N*	*%*	*OR (95% CI)*	*p*	*Chi-squared (X^2)*
Sex (Male / Female)	4645 / 4075	53.3 / 46.7	3053 / 3274	48.3 / 51.7	1.37 (1.24, 1.51)	<0.001	36.90
Being bullied (Yes / No)	592 / 2567	18.7 / 81.3	840 / 4132	16.9 / 83.1	0.97 (0.85, 1.10)	0.646	4.53
Childhood abuse (Yes / No)	563 / 5444	9.4 / 90.6	654 / 5192	11.2 / 88.8	1.26 (1.08, 1.48)	0.004	10.59
**17-18 years old time point**							
	**Non-participating group in the study**		**Participating group in the study**		**Non-participating versus participating**		
	Mean	*SD*	*Mean*	*SD*	*OR (95% CI)*	*p*	*t-test (t)*
Gestational age (in weeks)	37.90	6.42	39.43	1.85	0.98 (0.95, 1.03)	0.083	15.59
Family Adversity score	4.83	4.52	3.68	3.87	0.95 (0.93, 0.96)	<0.001	-13.27
Emotional Temperament score	39.68	8.50	39.14	8.42	0.99 (0.99, 1.03)	0.321	-3.19
	**Non-participating group in the study**		**Participating group in the study**		**Non-participating versus participating**		
	N	*%*	*N*	*%*	*OR (95% CI)*	*p*	*Chi-squared (X^2)*
Sex (Male / Female)	5587 / 4558	55.1 / 44.9	2111 / 2791	43.1 / 56.9	1.72 (1.57, 1.89)	<0.001	190.71
Being bullied (Yes / No)	812 / 3667	18.1 / 81.9	620 / 3032	17.0 / 83.0	1.04 (0.92, 1.18)	0.525	1.84
Childhood abuse (Yes / No)	743 / 6830	9.8 / 90.2	474 / 3806	11.1 / 88.9	1.25 (1.07, 1.46)	0.004	4.74

© 2021 Morales-Muñoz I et al. *JAMA Network Open.*

eTable 2. Differences in Sociodemographic and Clinical Variables Between Nonparticipants and Participants, Ages 22 to 24 Years

**22-23 years old time point**							
	**Non-participating group in the study**		**Participating group in the study**		**Non-participating versus participating**		
	Mean	*SD*	*Mean*	*SD*	*OR (95% CI)*	*p*	*t-test (t)*
Gestational age (in weeks)	38.10	6.02	39.50	1.80	1.02 (0.99, 1.05)	0.147	12.18
Family Adversity score	4.71	4.49	3.40	3.57	0.93 (0.91, 0.94)	<0.001	-13.53
Emotional Temperament score	39.57	8.51	39.19	8.37	1.00 (0.99, 1.01)	0.728	-1.97
	**Non-participating group in the study**		**Participating group in the study**		**Non-participating versus participating**		
	N	*%*	*N*	*%*	*OR (95% CI)*	*p*	*Chi-squared (X^2)*
Sex (Male / Female)	6668 / 5458	55.0 / 45.0	1030 / 1891	35.3 / 64.7	2.38 (2.15, 2.64)	<0.001	366.63
Being bullied (Yes / No)	1058 / 4702	18.4 / 81.6	374 / 1997	15.8 / 84.2	1.05 (0.92, 1.21)	0.453	0.01
Childhood abuse (Yes / No)	836 / 8292	9.1 / 90.9	281 / 2444	10.3 / 89.7	1.23 (1.04, 1.34)	0.044	7.79

© 2021 Morales-Muñoz I et al. *JAMA Network Open.*

eTable 3. Symptoms of Greatest Relevance for Borderline Personality Disorder, Psychosis, and Hypomania

Psychopathological measure	Rank of symptoms (first, second, third)	N (%)
Borderline Personality Disorder	1. Anger symptom	1557 (24.3)
	1. Impulsivity symptom	1437 (22.6)
	2. Affective instability symptom	1301 (20.3)
Psychosis	1. Auditory hallucinations	145 (2.9)
	2. Visual hallucinations	112 (2.3)
	3. Delusions of being spied on	26 (0.5)
Hypomania	1. Needs less sleep when in “high” state	2811 (83.4)
	2. Feels more energetic when in “high” state	2695 (80.0)
	3. Talks more when in “high state”	2705 (80.5)

© 2021 Morales-Muñoz I et al. *JAMA Network Open.*

eTable 4. Descriptive Values of Cognitive Measures in Psychopathological Outcomes

	BPD 11-12 years			Psychosis 17-18 years			Depression 17-18 years			Hypomania 21-23 years		
	Yes	No		Yes	No		Yes	No		Yes	No	
	Mean (SD)	Mean (SD)	t-test, p	Mean (SD)	Mean (SD)	t-test, p	Mean (SD)	Mean (SD)	t-test, p	Mean (SD)	Mean (SD)	t-test, p
Selective attention, 8 years	8.62 (2.24)	8.80 (2.28)	-1.46, 0.761	8.50 (8.87)	8.87 (2.28)	-2.03, 0.868	8.69 (2.27)	8.88 (2.28)	-1.67, 0.729	8.94 (2.29)	9.09 (2.62)	0.58, 0.166
Sustained attention, 8 years	7.06 (3.75)	7.76 (3.70)	-3.47, 0.084	7.49 (3.93)	7.81 (3.63)	-1.06, 0.202	7.20 (3.63)	7.88 (3.64)	-3.61, 0.351	7.49 (3.68)	7.71 (3.58)	-0.53, 0.402
Attentional control, 8 years	18.17 (1.75)	18.35 (1.53)	-2.09, 0.009	18.17 (1.70)	18.36 (1.55)	-1.50, 0.105	18.24 (1.65)	18.40 (1.45)	-2.18, <0.001	18.38 (1.59)	18.45 (1.42)	-0.38, 0.293
Working memory, 10 years	3.33 (0.86)	3.44 (0.83)	-2.54, 0.155	3.34 (0.93)	3.47 (0.85)	-1.81, 0.056	3.44 (0.81)	3.47 (0.85)	-0.60, 0.579	3.31 (0.93)	3.50 (0.83)	-1.91, 0.056
Inhibitory control, 10 years	11.82 (3.27)	12.09 (3.05)	-1.68, 0.037	11.60 (3.25)	12.06 (3.04)	-1.91, 0.112	12.19 (3.04)	12.03 (3.06)	1.05, 0.670	12.14 (2.99)	12.38 (2.84)	0.69, 0.687

BPD=Borderline personality disorder

© 2021 Morales-Muñoz I et al. *JAMA Network Open.*

eTable 5. Associations Between Dependent Variables in Path Analysis

BPD=Borderline personality disorder

**Dependent variables**	BPD symptoms at 11-12 years		Psychotic experiences at 17-18 years		Depression at 17-18 years		Hypomania at 22-23 years	
	β	p	β	p	β	p	β	p
BPD symptoms at 11-12 years	---------	---	0.06	<0.001	0.07	<0.001	0.07	<0.001
Psychotic experiences at 17-18 years			---------	---	0.15	<0.001	0.07	<0.001
Depression at 17-18 years					---------	---	0.03	0.020

© 2021 Morales-Muñoz I et al. *JAMA Network Open.*

eTable 6. Significant Direct Associations Between Covariates and Dependent Variables in Path Analyses

	BPD at 11-12y		Psychosis at 17-18y		Depression at 17-18y		Hypomania at 21-23y	
	β	p	β	p	β	p	β	p
**Sex**	------	------	0.02	<0.001	0.08	<0.001	-0.01	0.036
**FAI**	0.05	<0.001	0.03	<0.001	0.08	<0.001	------	------
**Gestational age**			-0.04	<0.001	------	------	0.03	<0.001
**Emotional temperament**	0.01	0.045	------	------	------	------	------	------
**Bullying**	0.05	<0.001	0.02	0.016	------	------	------	------

© 2021 Morales-Muñoz I et al. *JAMA Network Open.*

eFigure. Flowchart of Avon Longitudinal Study of Parents and Children Study Participants

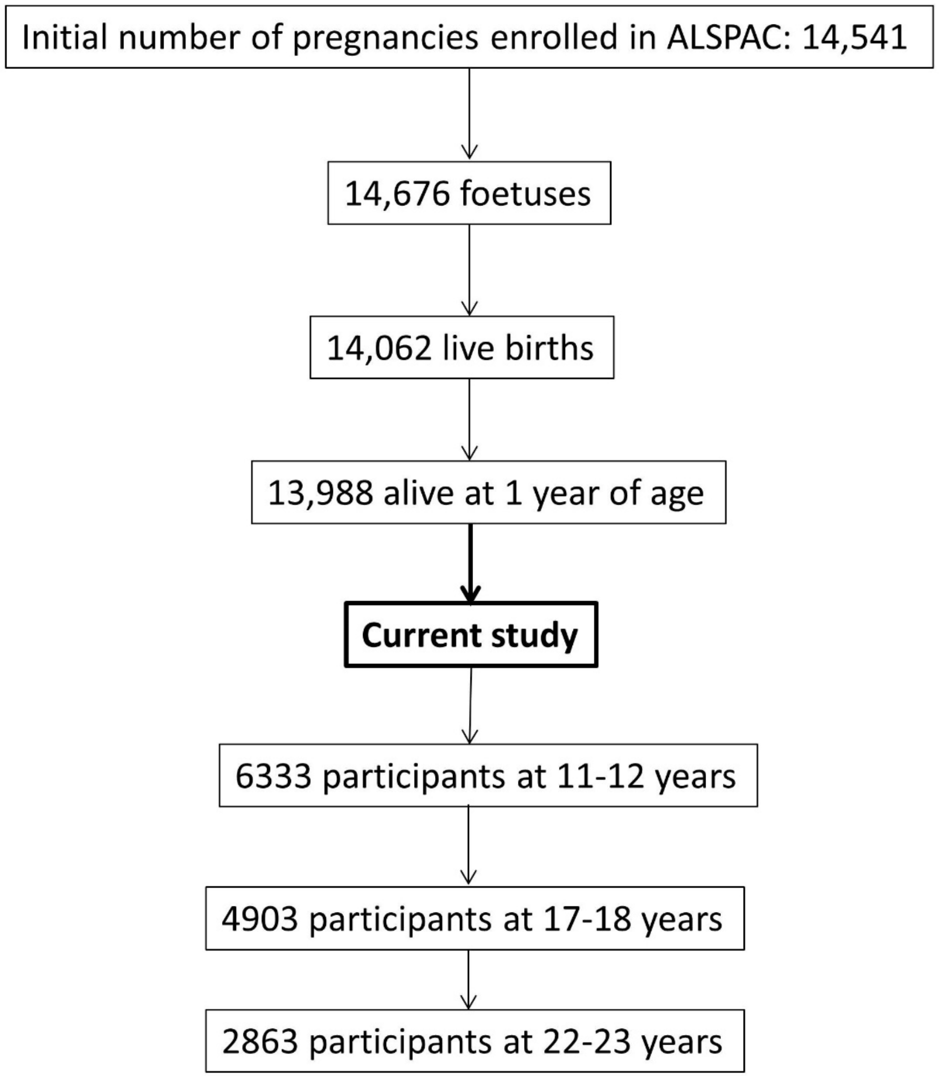

© 2021 Morales-Muñoz I et al. *JAMA Network Open.*
